# Henri Cochet's theory of angles in tennis (1933) reveals a new facet of anticipation

**DOI:** 10.1038/s41598-024-53136-7

**Published:** 2024-02-09

**Authors:** Nicolas Benguigui, François Rioult, François Kauffmann, Matt Miller-Dicks, Colm P. Murphy

**Affiliations:** 1 Normandie Univ, UNICAEN, CNRS, GREYC, Caen, France; 2 Normandie Univ, UNICAEN, CNRS, LMNO, Caen, France; 3 School of Sport, Health and Exercise Science, University of Portsmouth, Portsmouth, UK; 4 Cardiff School of Sport and Health Sciences, Cardiff Metropolitan University, Cardiff, UK

**Keywords:** Neuroscience, Psychology

## Abstract

In this study, we tested the theory of angles that was proposed almost a century ago by the tennis player Henri Cochet. This theory proposes that expert tennis players should position themselves on the bisector of the angle of the opponent’s possibilities in order to optimize shot return, suggesting a geometric occupation of the court relative to the opponent's affordances; namely what he/she is capable of doing. We tested this hypothesis by analysing player and ball positioning data from professional tennis matches recorded with a Hawk-Eye system. We compared this hypothesis with two alternative computational and probabilistic hypotheses which would consist in positioning oneself on the average or the median of the shots usually played from a given location. The results show that expert tennis players apply the principles of the theory of angles and thus confirm Henri Cochet's intuition. That is, for lateral court positioning, a geometric strategy is deemed optimal by expert players. It also appears that the more experienced the players are, the more precise their application of this strategy becomes.

## Introduction

A defining feature of expertise in fast-ball sports such as tennis and badminton is the requirement for players to position themselves so as to effectively cover and defend the playing area, relative to their opponent’s actions. The principles of positioning in these sports are codetermined by the spatiotemporal demands that characterize the game (e.g., size and surface of the field, properties of the ball), and the abilities of the competing athletes^1^. In the case of tennis, the challenge for players is to position themselves so that they can respond as effectively as possible to all potential actions of the opponent, with the probability of action outcomes varying based not only on player positioning^2^, but also respective player action tendencies^3^, abilities^4^, and the prior sequence of shots played within rallies^5^.

**Figure 1:**
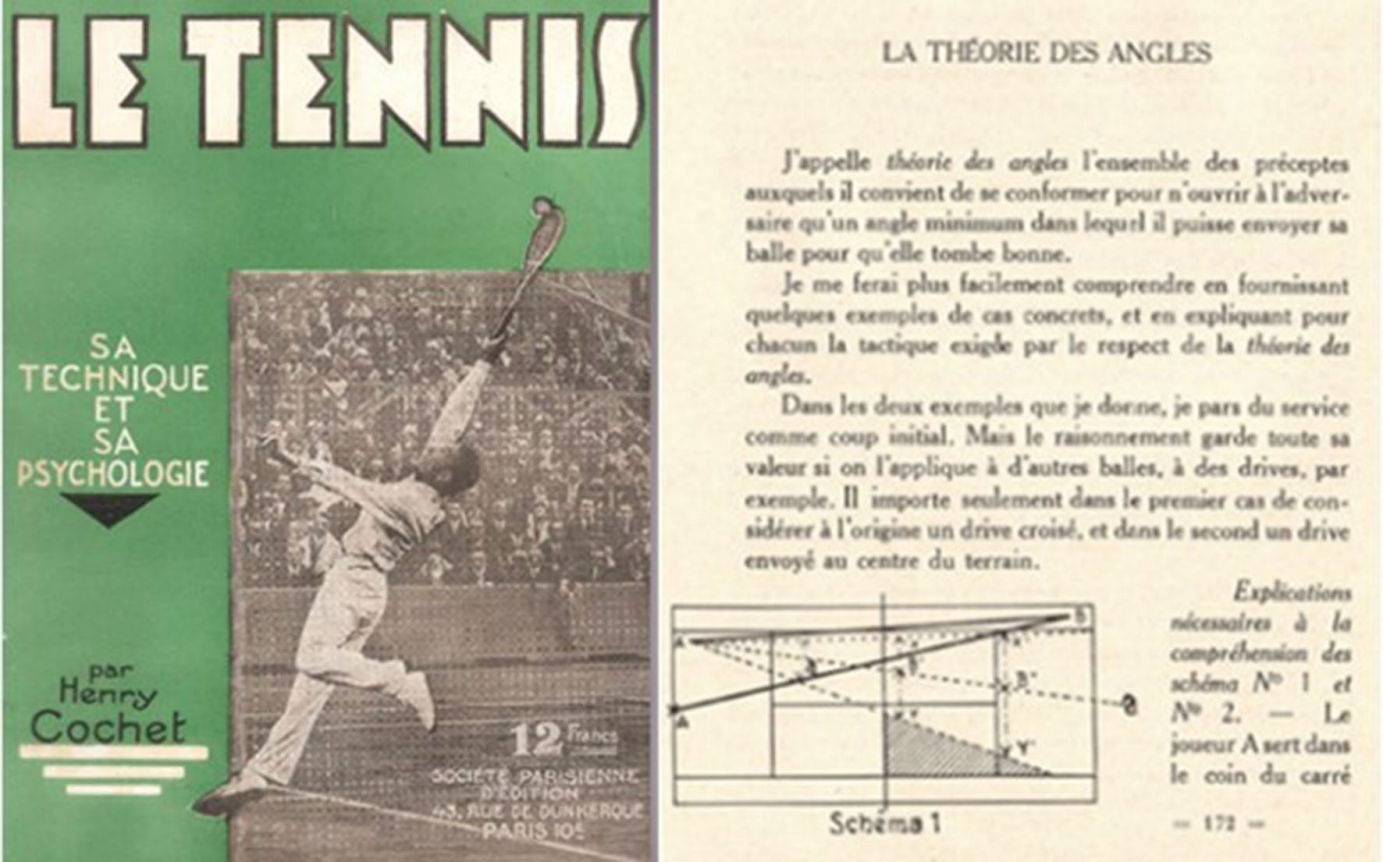
Cover and first page of the chapter on the “theory of angles” in Henri Cochet's book «Le tennis: sa technique et sa psychologie» (Tennis: its technique and psychology) (1933).

**Figure 2:**
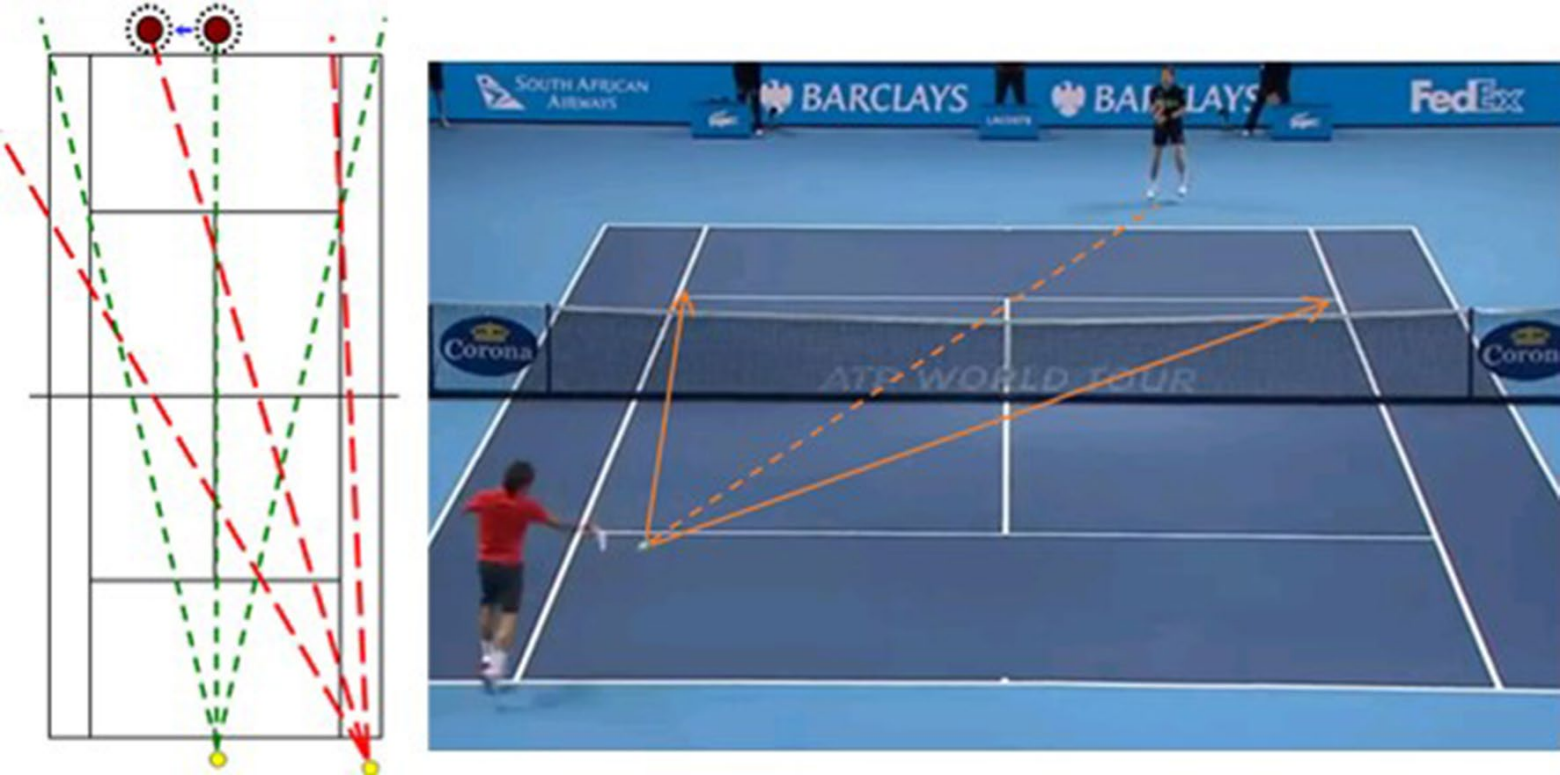
Illustration of the "theory of angles". On the left are hypothetical angles formed by the possible trajectories from two shots played from different positions on the court. The yellow discs represent the ball. Green and red lines denote possible trajectories and bisectors when the ball is struck from a central and off-centre court position, respectively. Cochet^6^ proposed that the bisectors should reflect a player’s optimal court positioning (brown discs) prior to the ball being struck. Critically, when the opponent is off-center, the angle formed in red defines a bisector which differs to when they are centrally positioned, creating a situation in which the optimal position for the receiving player is on the opposite side of the court. On the right is an image that illustrates the theory of angles unfolding in a game.

One of the long-held propositions for optimizing court positioning in tennis is the so-called "theory of angles", which was formalized by Cochet^6^ in order to explain the lateral court positions that players should adopt during rallies. Henri Cochet (1901–1987) was one of the musketeers of the French tennis team that won the Davis Cup between 1927 and 1932. He was a multiple Grand Slam winner and a teacher and trainer of players and coaches for several decades who greatly influenced the development of tennis in France. In his book entitled "Tennis: sa technique et sa psychologie” (Tennis: its technique and psychology), Cochet^6^ proposed that, according to the theory of angles (pp. 172–177: Fig. 1), expert players position themselves on the bisector of the angle of the trajectories that their opponent can hypothetically enact. A key feature of this strategy is that when the player who is about to strike the ball is displaced laterally to one side of the court, the corresponding lateral court position that will most effectively facilitate retrieval of the next shot for the opposing player is on the opposite side of the court (Fig. 2).

Despite the theory of angles^6^ being formulated nearly a century ago, the central tenets of this proposal have never been tested in extant literature. However, it is noteworthy that, akin to Cochet’s proposal, contemporary accounts of expertise have highlighted the importance of the spatial distribution of players and the significance of their relative positions on performance outcomes in team sports (e.g.^7,8^). Furthermore, and specific to the study of tennis, Palut and Zanone^9^ applied dynamic systems theory to study the lateral movements of tennis players. The researchers were able to categorize two dominant patterns of play, which captured the coordination dynamics of the two players as oscillators; one in-phase (where players moved in the same direction) and the other anti-phase (where players moved in opposite directions), showing that players’ movements were coupled to one another and were dependent on the shot(s) played in the rally (see also^10^). The authors suggested that this synchronization between players represented stabilization in the dynamical system, with shifts between in-phase and anti-phase thought to reflect the geometric evolution of the relationship between the players during the rally. Clearly, the findings of Palut and Zanone^9^ suggest that tennis players employ movement strategies that allow them to maintain a degree of stability within rallies (see also^11^). However, at a tactical level, the researchers did not specifically investigate how players position themselves relative to one another to effectively cover the court and defend against the opponent’s actions, which is a central facet of Cochet’s^6^ proposal.

Extensive literature studying anticipation in sports has shown that, to judge opponent intentions, experts are capable of generating and utilising tactical information that unfolds during the game^12^ in tandem with movement kinematic information from an opponent's action (e.g.^13^). Triolet et al.^14^ showed that experts, specifically in tennis, do not anticipate (i.e., base their responses on information picked up prior to the opponent striking the ball) as frequently as perhaps first believed (e.g.^15^). This can be observed in the imprecise nature of *early* anticipation, which can lead to players being deceived^16,17^. Alternatively, it has been proposed that players may adopt a conservative strategy that increases the likelihood of moving in the correct direction, rather than committing to a response too early when anticipating an opponent’s action^14^.

When considered relative to extant perspectives in the literature, the theory of angles^6^ offers a new way of thinking about expert sports anticipation. Specifically, rather than anticipation being a case of predicting what an opponent *will do*, when couched in terms of Cochet’s theory of angles, anticipation becomes a case of positioning oneself to be responsive to what an opponent *can do*. That is, anticipation reflects the perception of an opponent’s opportunites for action, a proposal that aligns with Gibson's theory of affordances^18^. Whilst the study of affordance perception in sports expertise has largely examined what the environment offers an athlete relative to her or his action capabilities^1^, research efforts have begun to show that expert athletes are sensitive to the affordances offered by the environment for another person’s actions^19^. Therefore, consistent with such evidence, Cochet’s novel proposal is that positioning on the court is based on a perception of the unfolding game situation and the action capabilities of the opponent (see also^20^).

The theory of angles^6^ could be challenged by alternative hypotheses. Indeed, expert players may tend to move towards a lateral position that reflects the central tendency of the distribution of previous shots hit by his/her opponents from the area of the court from which they are about to strike the ball. This strategy would mean that player positioning is determined by the average trajectory of the previous shots played by their opponents. Thus, assuming that opponents were to act consistently with their prior actions, this strategy of positioning oneself on the mean or median of trajectories originating from a specific area of the court could be conceptualized as anticipation of what the opponent *will probably do*. It should be noted that this strategy would be less physically demanding than the geometric strategy proposed by Cochet^6^ because it would minimise the amplitude of the actions needed to reach each shot. This type of strategy based on central values of the shot distribution would be compatible with research demonstrating the ability of experts to pick up and integrate probabilistic information when judging the outcome of upcoming events (e.g.^2,3,21,22^).

To shed new light on these potential explanations of expert anticipation, examination of world-class tennis players’ actions and court positions (e.g., Fig. 2) could be studied empirically. The aim of the current study was therefore to test the two proposals of how expert athletes position themselves spatially in order to anticipate what the opponent *can do* or *will probably do*: (1) the theory of angles, which corresponds to a geometric strategy, and (2) a probabilistic strategy that would correspond to the central values of the previous distribution of opponents’ shots. We further subdivide the latter strategy into the mean of the previous distributions (2a) and the median of the previous distributions (2b). This distinction is made due to the computational demand of calculating an optimal court placement based on the average of the previous (2a) or the centre of the previous shots (2b). To test these hypotheses, we used a novel dataset derived from a Hawk-Eye system, which is a multiple camera system that digitally reconstructs the positions of the ball and the players during the rally. In addition, we extracted the lateral movement speed of the players at the moment of the ball strike in m/s, in order to test the hypothesis that the further away the players were from their target replacement zone, the faster they would move to get closer to it.

## Methods

In order to address the question of the use of the theory of angles in world-class tennis, this study was based on the analysis of a unique and extensive dataset of 5679 tennis shots that were recorded using the Hawk-Eye system (Hawk-Eye Innovations Ltd., Basingstoke, United Kingdom), derived from competitive matches from the AEGON Championships at the Queen’s Club, London, played on grass courts. These matches involved 23 different professional players, all right-handed, who were ranked in the top 100 players in the world (*M*_*age*_: 27 years, *SD*: 3.37) (see Table 1 for individual data on age, highest ranking, number of matches played and won, and number of shot situations or occurrences analysed).

**Table 1:** Individual statistics and results for the 23 players with their age at the moment of the tournament, the highest ranking reached in their careers, the number of matches played as a professional player, and the percentage of matches won (Association of Tennis Professionals data in 2020).

	Player information					Algebraic values (CE)			Absolute values (AE)		
	Age in years	Best ATP ranking	Total matches	Percentage of wins	Number of occurrences analysed	Distance to the bisector	Distance to the mean	Distance to the median	Distance to the bisector	Distance to the mean	Distance to the median
Player 1	32.0	35	373	41.0	176	− **0.12**	0.22	0.47	0.85	**0.83**	1.10
Player 2	24.1	43	184	47.3	213	− 0.28	**0.11**	0.44	0.85	**0.81**	1.09
Player 3	27.8	4	956	65.5	225	− 0.20	**0.08**	0.30	**0.80**	0.83	1.18
Player 4	24.8	3	698	65.5	370	− **0.12**	0.28	0.66	**0.84**	0.91	1.28
Player 5	24.8	40	162	43.2	80	**0.03**	0.30	0.61	**1.19**	1.24	1.58
Player 6	24.8	4	572	71.7	504	− **0.04**	0.32	0.65	**0.90**	0.95	1.29
Player 7	22.1	3	445	61.6	233	− 0.12	**0.03**	0.28	**1.01**	1.02	1.35
Player 8	28.4	29	258	45.7	274	**0.12**	0.53	0.83	**0.92**	1.05	1.44
Player 9	23.1	41	80	42.5	222	− 0.41	− **0.10**	0.16	1.20	**1.14**	1.30
Player 10	32.3	1	878	70.2	567	− **0.01**	0.35	0.70	**0.897**	0.900	1.20
Player 11	21.1	53	94	34.0	72	− **0.11**	0.27	0.56	**1.10**	1.17	1.58
Player 12	31.4	37	391	44.5	106	− **0.20**	0.22	0.60	0.92	**0.88**	1.12
Player 13	33.1	19	563	52.0	213	− **0.14**	0.31	0.75	**0.86**	0.87	1.23
Player 14	28.2	39	158	39.9	90	− **0.03**	0.37	0.65	**0.82**	0.89	1.30
Player 15	26.1	1	839	78.1	709	− **0.04**	0.30	0.60	**0.84**	0.99	1.32
Player 16	23.1	39	123	42.3	120	**0.39**	0.80	1.07	**1.11**	1.32	1.67
Player 17	25.7	11	600	55.3	210	− **0.10**	0.32	0.74	0.97	**0.94**	1.25
Player 18	29.6	35	209	40.2	110	**0.21**	0.65	0.96	**0.77**	0.86	1.21
Player 19	30.1	60	224	33.9	226	**0.03**	0.42	0.85	**0.89**	0.93	1.29
Player 20	28.2	29	284	44.4	150	− **0.06**	0.20	0.49	**0.81**	0.82	1.10
Player 21	26.2	52	140	35.7	79	**0.05**	0.32	0.59	**0.92**	0.95	1.14
Player 22	28.2	5	627	68.6	469	− **0.14**	0.21	0.59	**0.970**	0.974	1.25
Player 23	26.3	89	82	30.5	261	− **0.10**	0.23	0.45	**0.94**	1.00	1.28
**Mean**	**27.0**	**29.2**	**388.7**	**50.2**	**246.9**	− **0.06**	**0.29**	**0.61**	**0.93**	**0.97**	**1.29**
**Standard error**	**3.27**	**23.0**	**275.6**	**14.0**	**169.7**	**0.17**	**0.19**	**0.21**	**0.12**	**0.14**	**0.15**

The algebraic and absolute values (Constant Error [CE] and Absolute Error [AE]) correspond to the mean distance to the predicted value based on the bisector, mean and median. The minimal values for the predictors are indicated in bold.

*M* mean, *SE* standard error.

In taking part in the tournament, players agreed to have their data collected by the Hawk-Eye system which is used for refereeing assistance and computer graphics for television broadcasts. The tournament organizers had the rights to access and use the data for research and provided the data to the authors of this study in this context. The rights to use the data implied that the anonymity of the players would be respected at all stages of the processing and publication of this study, and would not be lifted under any circumstances. The work was carried out according to the ethical guidelines of the lead university.

The Hawk-Eye system used to collect the data (Hawk-Eye Innovations Ltd., Basingstoke, United Kingdom) comprised of 10 cameras placed around the court, which sampled at 50 Hz, recording the 3D positions of the ball and 2D positions of the players (see^23^ for earlier research using this data). From these data, the positions of the ball and the players in 2D (x and y coordinates corresponding to the width and depth of the court) were recorded at the time of ball-strike during all back-court rallies. The analysis focuses on back-court situations where both players were positioned behind the service boxes and the ball was struck after a bounce (i.e., not by a volley). This criterion was developed because when players are positioned closer to the net, they are often not able to reposition themselves effectively on the court (see^14^). For the same reason, serves, returns of serves, and the last shot of each rally were excluded. This resulted in a total of 5679 shot situations or occurrences, 247 on average per player (range: 72–709).

**Figure 3:**
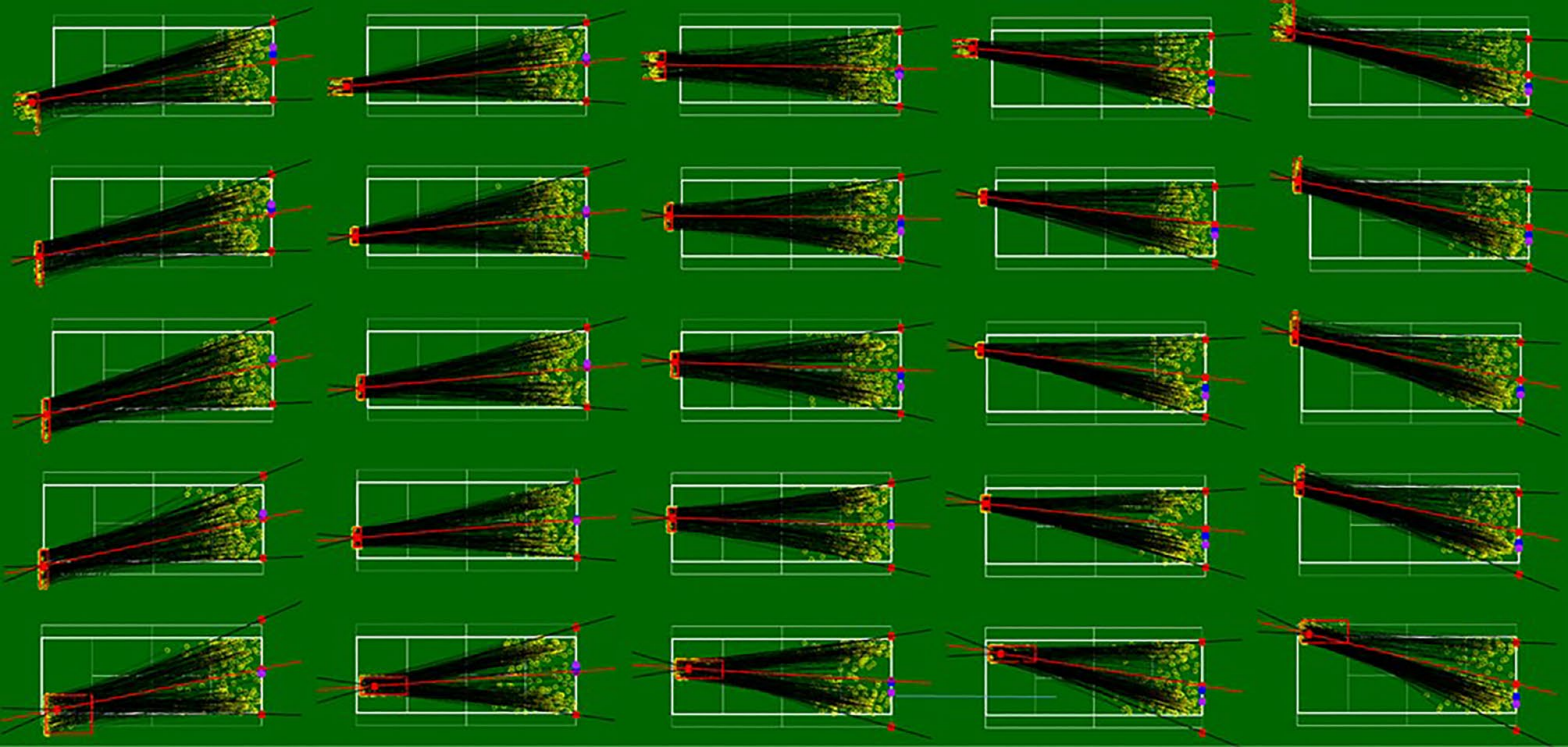
A representation of all 25 shot zones with the set of shots, the mean point summarising these shots and the predictions for the mean, median and bisector of the angle of the possible trajectories (see Fig. 4 for more details).

**Figure 4:**
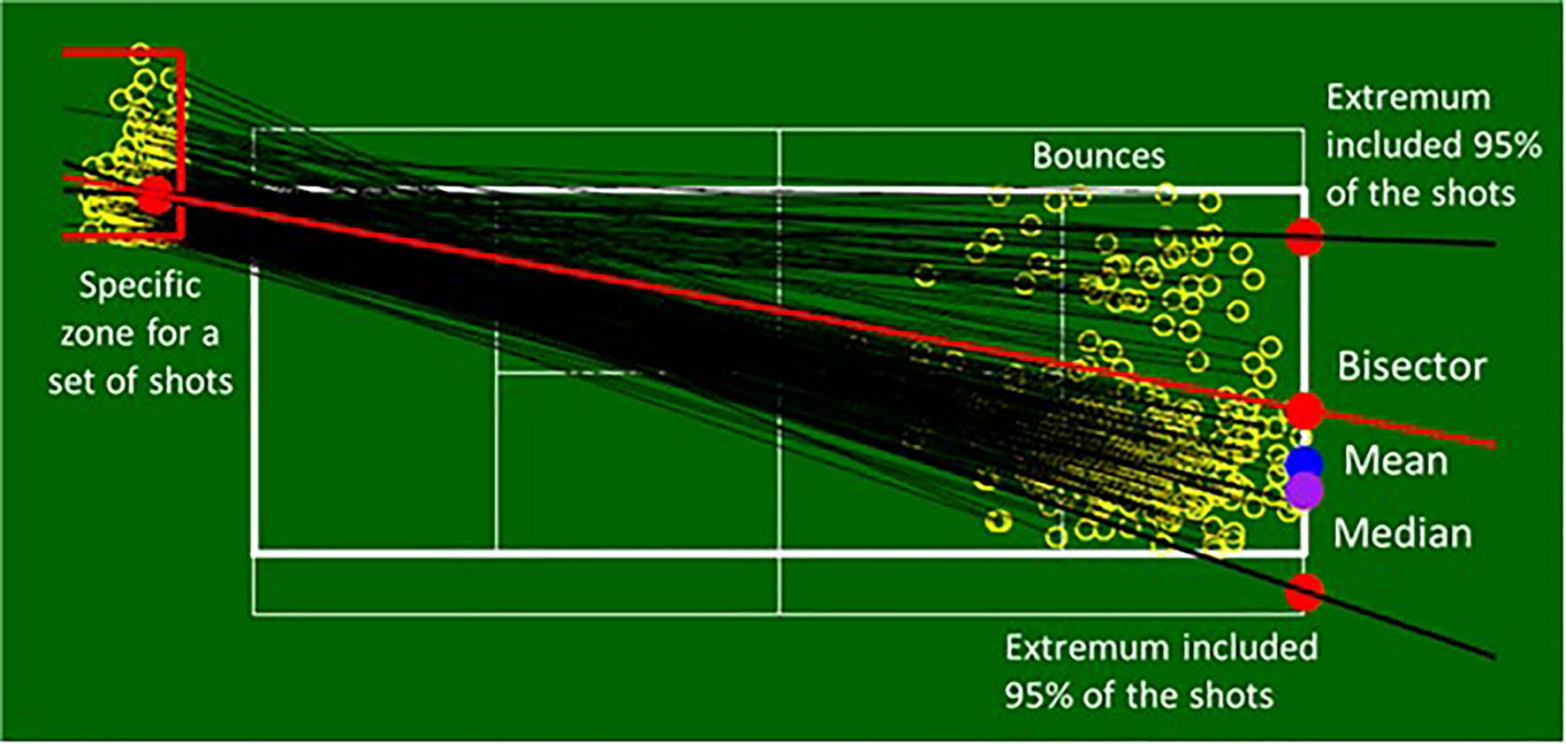
Illustration of a shot zone representative of the mean (blue disc) and median (purple disc) trajectories and the bisector (red line) of the angle of the possible trajectories (black lines) with the red discs reflecting this space and the bisector.

To identify the bisectors, means and medians of reference, and test our hypotheses, a 5 × 5 grid was applied to the tennis court in order to distribute the 5679 occurrences between 25 zones, with the same number of occurrencess in each zone (227), as shown in Fig. 3. For a given zone, the mean starting point of the ball was calculated and from this point, the mean of the trajectories produced and the median separating the distribution of trajectories were calculated. The angle of the possible trajectories was determined to include 95% of the shots, with the most extreme 5% (2.5% on each side) being discarded to avoid atypical shots modifying this angle too significantly. Once the angle was calculated, the bisector was calculated (Fig. 4).

**Figure 5:**
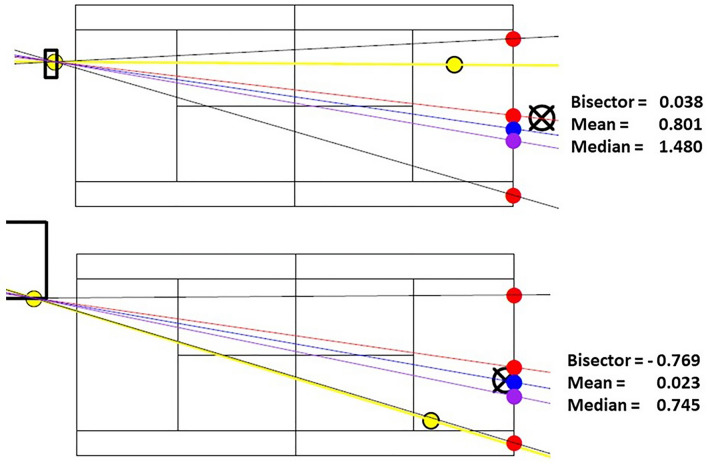
Two examples of distance calculations in meters from the receiver's position (cross circle on the right) to the bisector, mean and median values (red, blue, and purple discs, respectively). The position of the opponent's shot (yellow disc on the left), the ball trajectory produced (yellow line), and the impact in the receiver's field (yellow disc on the right) are also indicated.

In a second step, the position of the player on the lateral axis was measured at the moment of the opponent's strike in relation to the three predictions; (1) bisector; (2a) mean; and (2b) median, according to the procedure described in Fig. 5. This procedure was repeated for all the occurrences that had been extracted and made it possible to calculate an algebraic distance in metres (Constant Error: CE) and an absolute distance (Absolute Error: AE). In addition, in order to test the hypothesis that the further away the players were from their target replacement zone, the faster they would move, we extracted the lateral movement speed of the player at the moment of the strike in m/s. Based on this assumption, the aim was to correlate the movement speed with the distance to the replacement zone calculated from the three predictors (bisector, mean and median).

For CE and AE, we conducted ANOVAS with each of the three predictors: (1) bisector; (2a) mean; and (2b) median as repeated measures and Newman-Keuls post-hoc tests to differentiate between them in the case of a main effect. We further conducted regression analyses between distance to the predictor and lateral travel speed. Finally, for the best predictor identified from these analyses, we planned a multiple forward stepwise regression analysis to determine which individual factors would predict players' ability to accurately position themselves in the optimal location. The predictors were age, highest ranking, number of matches played on the professional circuit, and percentage of wins on that circuit as indicators of performance level.

## Results

The average distances in algebraic (CE) and absolute (AE) values are presented for each player in Table 1. For 19 of the players minimal values for CE were observed for the bisector predictor, while for the remaining four players minimal values for CE were observed for the mean predictor. The minimum values for AE were calculated for the bisector predictor for 18 players and the mean predictor for five players.

The repeated measures ANOVA revealed a significant main effect of predictor on CE (F(2,44) = 51.29, *p* < 0.05, ƞ^2^ = 0.71). A post hoc Newman–Keuls test revealed that CE was significantly smaller for the bisector (− 0.06 m) than the mean (0.29 m, d = 2.00), which was significantly smaller than for the median (0.61 m, d = 1.56). A t-test comparing each predictor to 0 revealed that the values for the mean and median were different from 0 (t(22) = 7.37, *p* < 0.05, d = 2.17, and t(22) = 13.76, *p* < 0.05, d = 4.06, respectively) while the values for the bisector were not significantly different from 0 (t(22) =  − 1.79, *p* > 0.05, d = 0.53).

ANOVA revealed a significant main effect of predictor on AE (F(2,44) = 247.10, *p* < 0.05, ƞ^2^ = 0.92). A post hoc Newman-Keuls test showed that AE was smaller for the bisector (0.93 m) than the mean (0.99 m, d = 0.30), which was smaller than for the median (1.29 m, d = 2.16).

**Figure 6:**
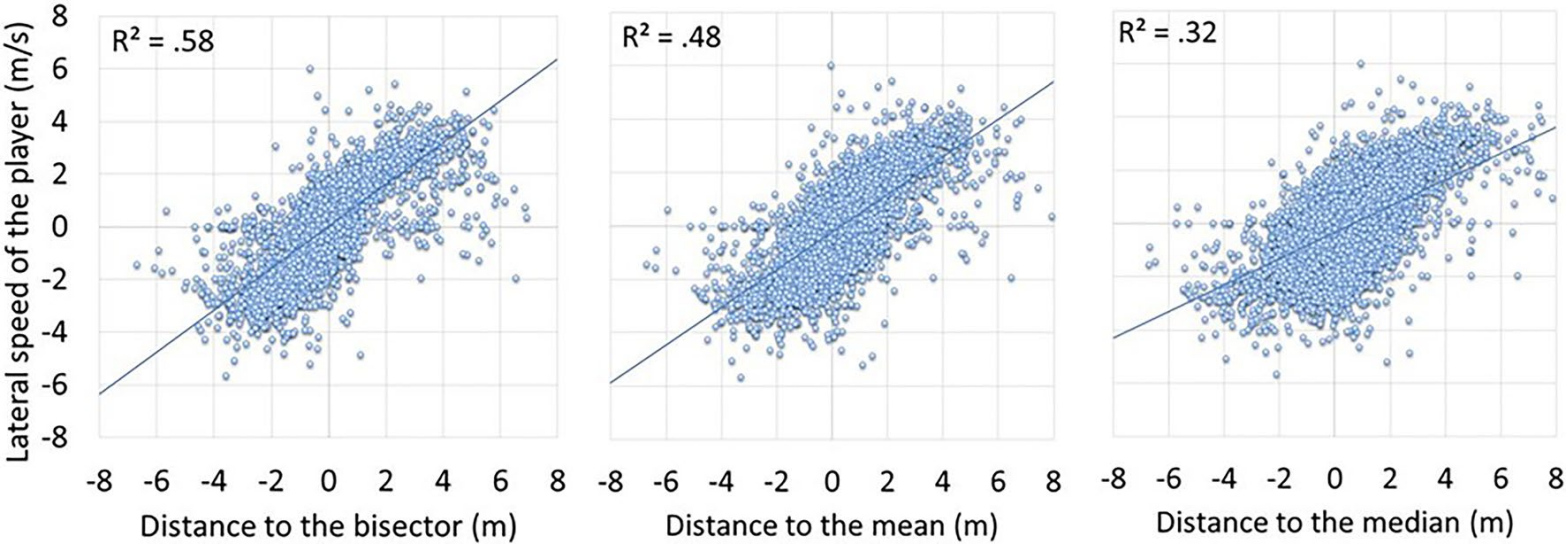
Linear regression showing the relationship between the lateral speed of the receiver and the distances to the bisector (left), mean (middle) and median (right), respectively.

To test the hypothesis that the further away the players were from their target replacement zone, the faster they would move to get closer to it, regression analyses between distance to the possible targets for replacement (bisector, mean, median) and lateral speed of the player at the time of the opponent's shot showed that the strongest correlation was for the bisector predictor with an R^2^ of 0.58 compared to 0.48 and 0.32 for the mean and median predictors, respectively (Fig. 6; the three correlations were significant with values of F(1,5978) = 8307.54; 5451.90; 2760.57, respectively).

To determine whether player variables predicted the distance from the bisector of the angle of possible trajectories, we used a forward stepwise multiple regression with AE as the dependent variable and age, highest ranking, number of matches played on the professional tour, and winning percentage as independent variables. In the first step of the analysis, age appeared as the best predictor (F(1, 21) = 10.28, *p* < 0.05) with 33% of the variance explained (R^2^ = 0.33). This is a negative relationship which shows that the older the player, the smaller the distance to the bisector. In the second and final step of the multiple regression, the number of matches played on the main professional circuit appeared to explain an additional 6% of variance. The final predictive equation with a β of − 0.017455 for the age of the players and a β of − 0.000117 for the number of matches played thus explained 39% of the variance (F(2,20) = 10.30, *p* < 0.05). Note that for the number of matches played, the simple regression was also negative, showing a decrease in the distance to the bisector as the number of matches played increased (R^2^ = 0.18).

## Discussion

The aim of this study was to test the theory of angles proposed by Cochet^6^, which stipulates that to facilitate an effective response to an upcoming shot, expert tennis players position themselves on the bisector of the angle of the possible trajectories that the opponent can produce. This novel research question was formalised as a hypothesis of geometric occupation of the court resulting from a player’s anticipation of the opponent's affordances in terms of the possible trajectory of shots that could be played. We compared this hypothesis to potential alternative strategies based on the use of central values of the distribution of shots played, i.e., the mean or median, involving computational and probabilistic principles. The extensive dataset included in the novel analysis made it possible to compare these proposals from the observed positions of the players at the moment the ball was struck.

The results directly support Cochet's hypothesis^6^. During back-court exchanges, to effectively respond to the opponent’s shot, the expert tennis players were found to position themselves at the bisector of the angle of possible trajectories. At the moment the opponent struck the ball, the distance players positioned themselves from the bisector was the smallest, both in terms of algebraic and absolute values, thus reflecting the players' search for this court position (Table 1). Moreover, the algebraic value measured for the bisector was not statistically different from 0, reflecting that, on average, players behaved in line with this prediction. This result was confirmed by the analysis of the lateral displacement speeds of players at the moment the opponent’s shot was played, which was better predicted by the distance to the bisector than by the mean and the median (Fig. 6). Expert tennis players appear to move and reposition themselves more quickly the further they are from the zone corresponding to the bisector of the angle of possibilities, indicating their intention to position themselves there to best respond to the opponent’s upcoming shot.

A striking result from the current study is the minimisation of the distance to the bisector as a function of the age and experience of the respective players. It appears that experience in a population of world-class tennis players leads to increasing accuracy in the perception of possible shot trajectories. It may be deemed surprising that indicators of playing level such as the best ranking achieved, or the percentage of matches won, did not account for any significant proportion of the variance. However, it should be noted that the number of matches played on the main circuit, which was included in the predictive equation, is an indicator of the level of play, both for the duration of a career and for the number of matches won. In this respect, the number of matches played was strongly correlated with the best world ranking achieved (R^2^ = 0.71) and winning percentage (R^2^ = 0.79), based on linear regressions between these variables. Therefore, the distance players positioned themselves from the bisector is also an indicator of performance that can potentially distinguish between world-class tennis players. Placing oneself as close as possible to the bisector of the angle of possible trajectories contributes to tennis performance through better occupation of the court^20^. This novel finding suggests that there is scope for training or awareness-raising on these issues, or for testing to determine the effectiveness of players’ positioning strategies.

Cumulatively, our findings suggest that expert tennis players employ the more conservative of the positioning strategies we compared^4,14,24^. By placing themselves on the bisector of the angle of possibilities, players maintain an equal opportunity of reaching the most extreme shots that can be delivered by their opponents to either side of the court. This positioning is to the detriment of a probabilistic strategy that would likely reduce the requisite displacement to the ball but potentially expose the player to shots played on the open side of the court. This can be seen for example in Fig. 4, where a placement on the mean or median trajectory would considerably open up the court on the right side. Moreover, a strategy based on central values such as the mean or median of previous shot trajectoires and probable outcomes, could be too demanding, in terms of the computational and memorization processes involved^25^. It is therefore the geometric strategy relating to the *possibilities*, rather than the *probabilities*, of the opponent that appears to be preferred, providing support for a sharpened capacity to perceive the opponent's affordances^18–20^.

One of the questions that remains unanswered from this research is whether expert performers may employ a combination of these strategies. While the geometric strategy seems to be largely dominant, it is possible that it is weighted by a probabilistic strategy leading to some adjustments being made in certain instances. It is then possible that individual players may modulate the implementation of this strategy relative to the situation they are in or to their preferences. For example, a player who feels more comfortable playing forehands might tend to position themselves more closely to the backhand side because they prefer to protect this side of the court or they may know that they can cope with longer displacements on the forehand side because of their skill level playing this shot. Moreover, these data may represent strategies that are employed in more neutral situations in which players are neither attacking nor defending. A more probabilistic strategy may be employed in defensive situations when anticipating what the opponent will do is necessary, due to the extreme time constraints involved^14^.

Whilst the novel data and analyses presented in the current study were obtained from 23 world-class tennis players, the data were collected from one international grass-court tennis tournament. Therefore, future work would benefit from building on these findings, including, where possible, a larger sample size collected across more international tournaments to further examine Cochet’s theory. However, based on existing research^14^, it is likely that this principle of court occupation would be observed regardless of playing surface, and by all world-class tennis players. Moreover, future work may also look to extend the current findings through qualitative and experimental methods to further extend the application of affordance perception to the study of expertise^26^. For instance, it is possible that the general strategy we observed in this study could also be modulated by knowledge of a particular opponent’s action preferences. Indeed, at the professional level, some players have the opportunity to face the same opponent several dozen times during their career. Moreover, players can also acquire knowledge of their opponent’s preferences through observation of video footage. It is thus possible that these repeated encounters or video analysis of the opponent’s strategy facilitate the development of more specific strategies that take into account individual opponent action preferences. This would mean that affordance perception as observed in the current work could be modulated by cognitive declarative memory processes about the opponent^26,27^. This could result in very specific positioning strategies that were not captured in our study. However, one can suppose that such behaviour is likely limited to very specific situations and that our study clealrly shows the most typical strategy.

## Conclusion

In this study, we conducted a novel analysis of a unique dataset from professional tennis players, to verify Cochet's^6^ theory of angles. The current findings suggest that the theory of angles strategy corresponds well to the behaviour of world-class tennis tennis players, as they seek to occupy and effectively defend the court. This strategy, which is based on a geometrical perception of the opponent’s possibilities of play, can be defined as a form of anticipation which minimises risk based on perception of the opponent's affordances. This novel study opens the door for future applied analyses to identify preferences of specific players, identify strengths and weaknesses of players, and design training protocols to develop these skills.

## Data Availability

The datasets generated during and/or analyzed during the current study are available from the corresponding author on reasonable request.
